# Proteomic analysis of the SIRT6 interactome: novel links to genome maintenance and cellular stress signaling

**DOI:** 10.1038/srep03085

**Published:** 2013-10-30

**Authors:** Federica Simeoni, Luisa Tasselli, Shinji Tanaka, Lidia Villanova, Mayumi Hayashi, Kazuishi Kubota, Fujio Isono, Benjamin A. Garcia, Eriko Michishita-Kioi, Katrin F. Chua

**Affiliations:** 1 Department of Medicine, Division of Endocrinology, Gerontology and Metabolism, School of Medicine, Stanford University, Stanford, California 94305, USA; 2 Geriatric Research, Education, and Clinical Center, VA Palo Alto Health Care System, Palo Alto, California 94304, USA; 3 Frontier Research Laboratories, Daiichi Sankyo Co., Ltd., 1-2-58 Hiromachi, Tokyo 140-8710, Japan; 4 Department of Experimental Medicine, Sapienza University, Rome, Italy; 5 Discovery Science and Technology Department, Daiichi Sankyo RD Novare Co., Ltd., 1-16-13, Kitakasai, Tokyo 134-8630, Japan; 6 Department of Biochemistry and Biophysics, University of Pennsylvania, Philadelphia, PA 19104, USA; 7 Current address: Venture Science Laboratories, Daiichi Sankyo Co., Ltd., 1-2-58 Hiromachi, Tokyo 140-8710, Japan.

## Abstract

The chromatin regulatory factor SIRT6 plays pivotal roles in metabolism, tumor suppression, and aging biology. Despite the fundamental roles of SIRT6 in physiology and disease, only a handful of molecular and functional interactions of SIRT6 have been reported. Here, we characterize the SIRT6 interactome and identify 80+ novel SIRT6-interacting proteins. The discovery of these SIRT6-associations considerably expands knowledge of the SIRT6 interaction network, and suggests previously unknown functional interactions of SIRT6 in fundamental cellular processes. These include chromatin remodeling, mitotic chromosome segregation, protein homeostasis, and transcriptional elongation. Extended analysis of the SIRT6 interaction with G3BP1, a master stress response factor, uncovers an unexpected role and mechanism of SIRT6 in regulating stress granule assembly and cellular stress resistance.

SIRT6 is a chromatin regulatory factor in the sirtuin family of enzymes, whose members have central roles in aging, metabolism, and cancer biology. SIRT6-deficiency in mice leads to severe metabolic defects, genomic instability, and accelerated tumorigenesis^1^,^2^,^3^,^4^. Conversely, over-expression of SIRT6 can extend mouse lifespan^5^. SIRT6 is an NAD-dependent enzyme that selectively removes specific chromatin marks associated with epigenetic and gene-regulatory functions. It deacetylates lysines K9 and K56 on histone H3 (H3K9, H3K56), and multiple studies have demonstrated important functions of this chromatin regulatory activity in telomere maintenance, DNA repair, and transcriptional repression^2^,^3^,^6^. SIRT6 can also deacetylate non-histone proteins (DNA processing factor CtIP and acetyltransferase GCN5)^7^,^8^, mediate mono-ADP-ribosylation (of Parp-1 enzyme)^9^, and catalyze defatty-acylation (of TNF-α)^10^. Thus, SIRT6 coordinates numerous cellular pathways via distinct enzymatic activities.

Despite the fundamental roles of SIRT6 in physiology and disease, and our previous discovery that SIRT6 protein associates with multiple high molecular weight biochemical complexes^6^, only a handful of molecular and functional interactions of SIRT6 have been reported. Here, we present the first proteomic characterization of the protein interaction network of SIRT6, and identify 80+ previously unknown SIRT6-interacting proteins in multiple macromolecular complexes. The discovery of these SIRT6-associations considerably expands knowledge of the SIRT6 interactome, and suggests previously unknown molecular functions of SIRT6 in processes such as transcriptional elongation, chromatin remodeling, mitotic chromosome segregation, and protein homeostasis. Importantly, we have validated the power of this approach to identify physiologic functions of SIRT6, in an extended analysis of one of the most robust interactions of SIRT6 – an association with the master stress response factor G3BP1. In functional analysis of the SIRT6-G3BP1 interaction, we uncover a new role and mechanism of SIRT6 in modulating cellular stress resistance.

## Results

**Figure 1:**
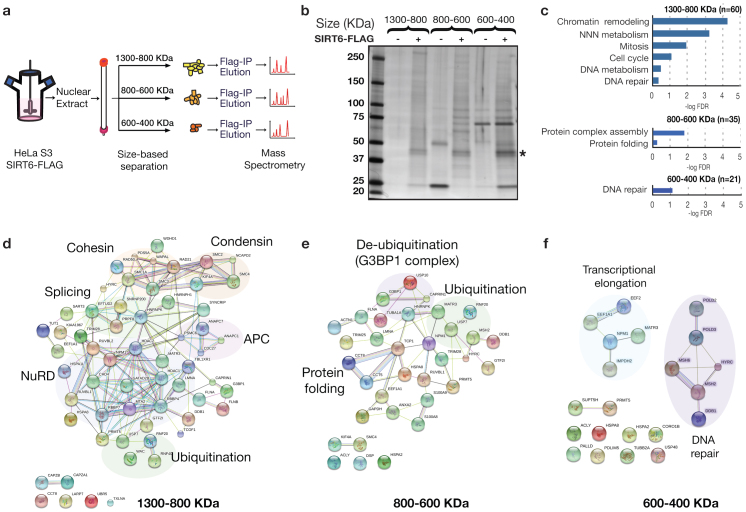
The SIRT6 interaction network. (a) Experimental approach used in this study. SIRT6 protein complexes were separated according to their size and subjected to proteomic analysis by mass spectrometry. (b) SDS-PAGE followed by silver staining of SIRT6 associated proteins isolated from the different size fractions of nuclear extracts (NE). Parallel IPs from control cells were performed as negative controls. *, SIRT6-FLAG. (c) Gene Ontology analysis of the proteins identified in the different fractions using the DAVID BP-Panther algorithm^24^. (d),(e),(f) STRING^11^ based reconstruction of protein complexes identified in the indicated size ranges. Shading indicates proteins belonging to a common cellular process.

In our strategy to enrich for physiologic SIRT6 interactions, we first generated HeLa S3 cells stably expressing SIRT6 with a C-terminal FLAG tag, and prepared nuclear extracts from large-scale cultures. Size-fractionation of the extracts by gel filtration showed that SIRT6-FLAG has similar size distribution as endogenous SIRT6 protein (Supplementary Fig. S1). To enrich for multi-protein complexes, we pooled gel filtration fractions corresponding to three large size ranges (400–600 KDa, 600–800 KDa, and 800–1200 KDa) and affinity-purified SIRT6-FLAG and associated proteins from these pools (Fig. 1a–c). Silver staining of the purified complexes revealed distinct patterns of associated proteins in the three size ranges (Fig. 1b). Liquid Chromatography-Mass Spectrometry (LC-MS) analysis identified over 80 proteins that were reliably enriched in the SIRT6-FLAG immunoprecipitates (IPs) in multiple independent experiments (Supplementary Table 1). The identified proteins included the known SIRT6-interacting protein DNA-PKcs (Fig. 1f, labeled “HYRC”)^6^, validating our approach. Importantly, a majority of the other identified proteins represent previously uncharacterized SIRT6 interacting factors. Of note, additional proteins identified in only one of our experiments are also potential candidates for novel physiologic SIRT6 interaction partners.

Gene Ontology (GO) analysis of the SIRT6-associated proteins detected in each of the size ranges (Supplementary Table 2), revealed enrichment for distinct functional GO categories. The top-ranked categories include chromatin regulation and DNA metabolism/repair, consistent with previously characterized functions of SIRT6. In addition, the analysis also identified numerous biological processes not previously linked to SIRT6, including mitosis/cell cycle regulation and protein folding/protein complex homeostasis (Fig. 1c).

To further elucidate the functional relationships between the SIRT6-interacting proteins and identify specific functional complexes, we mapped the proteins using the STRING interaction database (http://string-db.org/)^11^ (Fig. 1d–f). This analysis revealed multiple SIRT6-associated complexes that suggest previously unknown functional settings for SIRT6. These include components of the FACT complex that regulate transcriptional elongation, NurD complex involved in chromatin remodeling, cohesin and condension factors that regulate mitotic chromatin segregation, and diverse factors involved in protein homeostasis. Thus, our proteomic study considerably expands knowledge of the SIRT6 interactome and suggests testable hypotheses for elucidation of novel SIRT6 functions.

**Figure 2:**
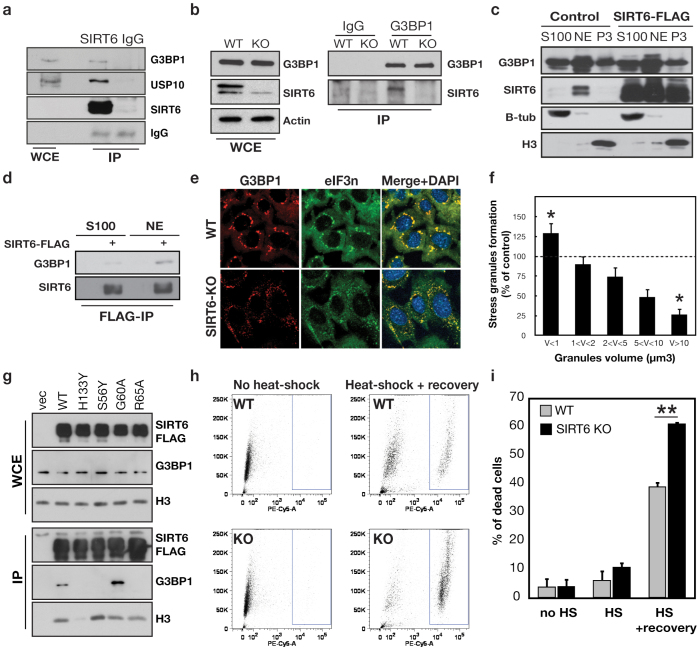
SIRT6 interaction with G3BP1 regulates stress granule assembly and cellular stress resistance. (a) Co-immunoprecipiation (IP) of endogenous SIRT6 and G3BP1 proteins from 293T cells. (b) Western analysis showing SIRT6 protein levels in wild-type and SIRT6 KO MEFs, and co-IP of endogenous SIRT6 and G3BP1 proteins from these cells. (c) Western analysis of SIRT6 and G3BP1 proteins in cytoplasmic (S100), nuclear extract (NE), and chromatin enriched (P3) biochemical fractions. Endogenous SIRT6 is detected in the Control samples (left). (d) Preferential interaction of G3BP1 and SIRT6 in the nuclear versus cytoplasmic fractions shown in (c). (e), (f) SIRT6 regulates SG assembly following heat shock (43°C, 90 min). SGs were detected by immunostain for G3BP1 and eIF3n. Stress granule volume (μm3) was quantified from confocal stacks of cells, using the ImageJ 3D counter plugin (see Methods). Granules were scored for volume range and number, normalized to total granule numbers. Error bars represent s.e.m. of 3 independent experiments. At least 100 cells were counted for each condition per experiment. (g) SIRT6 deacetylase, but not ribosylase, activity is required for interaction with G3BP1. Wild-type and mutant SIRT6-Flag proteins were IP'd from cells and levels of G3BP1 detected by western blot. The G60A mutant lacks ribosylase activity, R65A lacks deacetylase activity, and the H133Y and S56A mutants lack both. (h), (i) Increased cell death after heat-shock in SIRT6 KO MEFs. WT and SIRT6 KO MEFs were subjected to heat-shock allowed to recover for 24 h. Cell viability was quantified by propidium iodide staining and flow cytometry. Error bars represent s.e.m. of 3 independent experiments. For (f) and (i), the P value was calculated with the two-tailed Student's t-test. (*) indicates P < 0.05. (**) indicates P < 0.01. In all panels, western blots images shown are cropped to show the protein of interest, and all blots were performed under the same experimental conditions.

One of the most robust interactions that we identified in multiple independent experiments was between SIRT6 and G3BP1 (GTPase Activating Protein Binding Protein 1), a modulator of cellular stress resistance. G3BP1 is a fundamental component of cytoplasmic stress granules (SGs), which form in response to a variety of cellular stresses to mediate protective responses^11^,^12^. G3BP1 was reproducibly detected in both the middle and highest molecular weight SIRT6 complexes (Fig. 1d, e; Supplementary Table 2). Notably, we independently identified G3BP1 by LC-MS analysis of SIRT6-FLAG protein complexes isolated from two additional cell types (293T embryonic kidney and K562 erythroleukemia cells), and in purifications of proteins associated with the endogenous SIRT6 protein (data not shown). Western analysis confirmed the association of G3BP1 with Flag-tagged and endogenous SIRT6 in multiple cell types, including human 293T and HeLa cells, and mouse embryonic fibroblasts (MEFs) (Fig. 2a, b; Supplementary Fig. 2). G3BP1 was not detected in SIRT6 IPs from SIRT6-knockout (KO) MEFs^1^, validating specificity of the assay (Fig. 2b).

We also identified SIRT6 association with two other proteins that interact functionally with G3BP1 in SG assembly, USP10 and caprin-1^13^,^14^ (Fig. 1d, 1e, 2a). Analysis of these SIRT6 associations following siRNA knock-down of G3BP1, Caprin-1, or USP10 revealed that the association of SIRT6 with Caprin-1 and USP10 is mediated through G3BP1, whereas G3BP1 interacts with SIRT6 even in the absence of the other proteins (Supplementary Fig. 2a). The SIRT6-G3BP1 interaction was resistant to both DNAse and RNAse treatment, persists even under highly stringent conditions of 1 M NaCl, and was detected using recombinant proteins in vitro, arguing that the interaction is not bridged by additional factors (Supplementary Fig. 2, data not shown). Together, these observations suggest that G3BP1 is a robust and physiologic SIRT6-interacting protein.

The SIRT6-G3BP1 interaction was initially surprising to us, because SIRT6 is predominantly a nuclear, chromatin-associated protein^6^, whereas G3BP1 has been most studied in the context of cytoplasmic stress granule assembly^12^,^15^. We therefore used two independent biochemical fractionation assays to assess G3BP1 sub-cellular localization. As previously shown, endogenous SIRT6 protein is detected in either the soluble nuclear or chromatin-enriched fraction, depending on the fractionation protocol used^6^, whereas over-expressed Flag-tagged SIRT6 distributes in all three fractions (Fig. 2c, Supplementary Fig. 3). Unexpectedly, while endogenous G3BP1 is detected in the S100 cytoplasmic fraction, it is also detected at high levels in the nuclear and chromatin-enriched fractions that contain endogenous SIRT6 (Fig. 2c, Supplementary Fig. 3). Moreover, endogenous G3BP1 and SIRT6 co-fractionate in the same ~ 600 KDa size fractions of nuclear extracts, suggesting that they could form a physiologic, nuclear complex (Supplementary Fig. 1). Indeed, immunoprecipitation assays of SIRT6-FLAG from the different fractions revealed that the SIRT6-G3BP1 interaction occurs preferentially in the nuclear compartment (Fig. 2d).

We next sought to characterize the molecular mechanism through which the SIRT6-G3BP1 interaction could impact on stress granule assembly. Intriguingly, in co-IP assays of SIRT6 and G3BP1, we observed that multiple mutations in SIRT6 that inactivate its deacetylase activity dramatically reduce the interaction with G3BP1 (Fig. 2g). By contrast, a SIRT6 mutant protein that selectively inactivates the mono-ADP-ribosylase activity of SIRT6 (G60A) is fully proficient in binding G3BP1. These observations indicate that the deacetylase activity of SIRT6, but not its ADP-ribosyltransferase activity, is required for interaction with G3BP1. Notably, this dependence of the SIRT6-G3BP1 interaction on SIRT6 deacetylase activity was also observed for multiple other SIRT6-interacting factors identified in our studies (data not shown), and could reflect diverse mechanisms. One possibility is that these interactions depend on SIRT6 presence at chromatin, because the catalytic activity of SIRT6 is important for the protein's chromatin association^16^. In the case of G3BP1, however, the strength of the SIRT6-G3BP1 interaction does not correlate SIRT6 chromatin association for a panel of catalytically impaired SIRT6 mutants (Fig. 2g, compare H3 and G3BP1). A second model is that SIRT6 deacetylates G3BP1, which in turn stabilizes the interaction between the two proteins. Indeed, acetylation of G3BP1 on lysine K376 was recently reported^17^, and we observed reproducible, NAD-dependent deacetylase activity of SIRT6 on this residue, in *in vitro* peptide deacetylation assays (Supplementary Fig. 4). However, it was not possible to examine this deacetylase activity in cells, due to limited reagents for assaying this newly reported G3BP1 acetylation site. Nonetheless, our data are consistent with the model that G3BP1 is a novel SIRT6 substrate, and the deacetylase activity of SIRT6 stabilizes its interaction with G3BP1.

An important function of G3BP1 is to promote SG assembly in response to diverse exogenous stressors (e.g. heat shock, oxidative stress, or viral infection)^17^. In SG assembly, stalled translation complexes and RNA binding proteins are recruited to cytoplasmic inclusions, and this process is thought to protect cellular integrity by limiting accumulation of defective proteins^11^. Intriguingly, aberrations in the dynamics of SG assembly have been implicated in pathological protein aggregation observed in neurodegenerative disease and cellular senescence^18^,^19^. SG assembly initiates upon nucleation of core stress granule proteins early during stress. Then, under conditions of prolonged heat shock (> 60 min) or acute oxidative stress, SG assembly and maturation occurs with the fusion of small, nucleated granules into larger, bona fide SGs^18^,^19^.

Given the interaction of SIRT6 with G3BP1, we asked whether SIRT6 plays a role in SG assembly. We subjected SIRT6 KO and wild-type littermate control MEFs (See Fig. 2b), to prolonged heat shock (43°C for 90 min), and analyzed SG assembly by immunofluorescence stain of G3BP1 and another core SG component, translation initiation factor EIF3n. In the SIRT6 KO MEFs, there was a dramatic (~60%) reduction in the mature, large (volume > 10 um^3^) SGs, and a ~ 20% increase in very small (<1 μm3) granules (Fig. 2e,f). Similar results were observed in human U2OS cells in which SIRT6 was depleted by RNA interference (data not shown). These data suggest that SIRT6 is important for the assembly of mature SGs. Efficient SG dynamics is crucial for long-term cell survival after heat-shock^11^,^20^. We therefore asked whether the abnormal SG assembly in SIRT6 KO cells is associated with altered cellular stress resistance. The SIRT6 KO and WT control MEFs were subjected to heat shock, allowed to recover for 24 hours, and then cell viability analyzed by flow cytometry (Fig. 2h, i). Importantly, the SIRT6 KO MEFs underwent markedly increased cell death in response to this stress. Together, our results suggest a new role for SIRT6 in regulating SG formation and cellular stress sensitivity, through functional interplay with G3BP1.

## Discussion

In summary, we have combined biochemical complex purification and proteomic analysis to characterize a large-scale SIRT6 interaction network with more than 80 novel interactors. Our approach, using stably expressed epitope-tagged SIRT6 protein from purified HeLa cell nuclear extracts, combined with biochemical size-fractionation and affinity purification, has several advantages over previous attempts to identify SIRT6 interacting factors. By excluding the large amount of free SIRT6 protein from our purifications, our approach can minimize non-specific interactions and enrich for detection of physiologic interactions in the context of macromolecular complexes. Accordingly, our study uncovered previously unknown associations of SIRT6 with proteins involved in chromatin remodeling, mitotic chromosome segregation, DNA repair, and stress granule formation.

Our study also validates the power of this approach to identify novel and unexpected physiologic functions of SIRT6. Through our extended analysis of the SIRT6 interaction with G3BP1, we have discovered that SIRT6 is required for proper stress-granule assembly and resistance to cellular stress. Our data suggest that a nuclear interaction between SIRT6 and G3BP1 modulates cytoplasmic functions of G3BP1 in stress granule assembly. We provide evidence that this mechanism could be due to deacetylation of G3BP1 by SIRT6 in the nucleus, which could alter G3BP1 function upon cytoplasmic translocation. However, we cannot exclude that a minor fraction of SIRT6 present in the cytoplasm, as recently shown^10^, might directly regulate G3BP1 function during cytoplasmic SG assembly. Our data align well with recent studies showing that the *C. elegans* SIRT6 homolog, ceSir2.4, protects worms against various stresses including heat-shock^21^. Our results highlight the evolutionary conservation of a SIRT6 role in stress resistance, and suggest that at least part of SIRT6 function in these processes could be mediated via regulation of G3BP1 deacetylation and stress-granule assembly.

## Methods

### Cell culture

HeLa S3 cells stably expressing empty or SIRT6-FLAG from chromosomally integrated pBabe-puro were cultured in Minimum Essential Medium (MEM) Joklik modified for suspension culture and supplied with 5% newborn calf serum (NCS), penicillin, streptomycin and glutamate. Human Embryonic Kidney (HEK) 293 were maintained in Dulbecco's Modified Eagle Medium (DMEM) supplemented with 10% NCS, pyruvate, non essential amino-acid, penicillin, streptomycin and glutamate. Immortalized SIRT6 WT and KO mouse embryonic cell lines were grown in Advanced DMEM with 10% fetal bovine serum (FBS) and supplemented with penicillin, streptomycin, glutamate and beta-2-mercaptoethanol.

### Plasmids and siRNAs

Plasmids encoding empty and SIRT6-Flag pBabe-puro were previously described^6^. 3X-Flag-tagged SIRT6 mutants previously described^9^ were generated using the Quick Change Site directed Mutagenesis Kit (Stratagene). SiRNAs targetting human G3BP1, USP10, Caprin1 as well as the non-targeting control siRNA were obtained from ON-TARGETplus Smartpool siRNAs collection (Thermo Scientific).

### Antibodies

Antibodies directed against Human-SIRT6 were previously described^22^. Commercial antibodies used were: anti-G3BP1 for Western Blotting (A302-033A, Bethyl Laboratories); anti-G3BP1 for IF (ab56574, abcam), anti-USP10 (5553S, Cell Signaling Technology); anti-mouse-SIRT6 (ab62739, abcam); anti-eIF3n (Sc-16377, Santacruz), anti-Caprin1 (15112-1-AP, Protein Tech), anti-H3 (Covance), anti-beta-tubulin (05-661, Millipore), anti-actin (A5441, Sigma).

### Gel Filtration, immunoprecipitation and biochemical fractionation

SIRT6-associated protein complexes were purified from HeLa S3 nuclear extracts prepared as described previously^23^. 5 mg of enriched nuclear extract was loaded onto a 24-ml Superose 6 column (GE Healthcare) pre-equilibrated with a Hepes buffer (Hepes NaOH 25 mM pH 7.9, NaCl 150 mM, EDTA 1 mM, Tween20 0.1%), and ran in the same buffer at 0.5 ml/min. 0.5 ml fractions were collected and analyzed for the presence of SIRT6 or G3BP1 by western blotting. Molecular weight standards (GE Healthcare and Biorad) were used to calibrate the column.

SIRT6-associated protein complexes from fractions 1300-800, 800-600, and 600-400 KDa ranges were immunoprecipitated using anti-FLAG M2 monoclonal antibody conjugated agarose beads (Sigma). Immunocomplexes were eluted with Flag-peptide (Sigma), resolved on a 4–15% pre-cast SDS-polyacrylamide gel (BioRad) and stained using the SilverQuest silver-staining Kit (Invitrogen). When indicated, nuclear extracts were first incubated with DNAse and RNAse at a concentration of respectively 0.05 U/uglysate and 0.1 ug/ug lysate. FLAG-SIRT6 (from 293 cells), endogenous SIRT6 (from 293 cells) and G3BP1 (from MEF cells) were immunoprecipitated from lysate prepared in RIPA buffer (50 mM TrisHCl pH7.4, 150 mM NaCl, 2 mM EDTA, 1% NP-40, 0.1% SDS). Samples enriched for cytoplasmic, nucleoplasmic and chromatin fractions were prepared as previously described^24^.

### Sample preparation for mass-spectrometry

Affinity-purified proteins were precipitated twice with methanol-chloroform to remove the detergents and salts^25^. The precipitated proteins were collected by centrifugation and dried completely with a centrifuge evaporator. The dried proteins were dissolved with 8 M urea, 50 mM Tris-HCl, pH 8.0, 10 mM EDTA, pH 8.0, and 0.005% n-dodecyl-b-D-maltopyranoside (DM), and 10 mM DTT. The proteins were reduced at 37°C for 20 min, followed by alkylation by incubation at 25°C for 20 min in the dark with 20 mM iodoacetamide. The proteins were digested with 500 ng of trypsin (Modified trypsin, Promega) at 37°C for 12 h. The reaction was stopped by acidification with 5% formic acid to a pH lower than 2.5. Samples were desalted and concentrated by using slightly modified Stage Tips protocol^26^. Desalted peptides were dried with a centrifuge evaporator and dissolved with 8 μL of 5% formic acid.

### Protein identification and quantification by mass spectrometry

The LTQ-Orbitrap (Thermo Fisher Scientific) was equipped with an Agilent 1100 liquid chromatography system, which was modified to have a 200–300 nL/min flow rate by an in-house flow splitter. A homemade electrospray ionization tip column (100 μm internal diameter × 150 mm length) was packed with Inertsil ODS-3 C18 (3 μm, GL Sciences). Four microliters of the sample were injected to the LC-MS/MS system, and peptides were separated using a 95.5 min linear gradient of 5 to 28% acetonitrile in 0.125% formic acid. The LTQ Orbitrap was operated in data-dependent acquisition mode. Full MS scans (*m/z* range 350–1500) were acquired with a resolution of 60,000 in the Orbitrap analyzer. The 10 most intense ions were fragmented using collision induced dissociation and MS/MS spectra were acquired in the ion trap. All runs were performed in duplicates. Tandem mass spectra from raw files were extracted by a software suite for proteomics developed by Gygi lab from Harvard Medical School^27^ and submitted to Mascot program (Matrix Sciences) for database searching against the SwissProt human sequence database (Release: 2012_01 and 2012_03) supplemented with protein sequences from cRAP, a database of common contaminating proteins by the Global Proteome Machine Organization (http://www.thegpm.org/crap/index.html), using the following parameters; maximum missed cleavage: 1, static modification: carbamidomethylcysteine, variable modification: methionine oxidation and serine, threonine, and tyrosine phosphorylation, mass tolerances for precursor and fragment ions of 50 ppm and 0.8 Da, respectively. Peptide- and protein-level false discovery rates (FDRs) were filtered to 1% using the target-decoy strategy to distinguish correct and incorrect identifications^28^. To determine interacting proteins of SIRT6 that were enriched in FLAG-tagged SIRT6 in comparison with the negative control (NC), identified proteins were briefly divided into different levels based on peptide-spectrum matches (PSMs) from SIRT6 sample as follows: Level 1: PSM > 50, Level 2: 50 ≥ PSM > 20, Level 3: 20 ≥ PSM > 10, and Level 4: 10 ≥ PSM > 5. Subsequently, we defined 2-, 3-, 4-, and 5-fold enrichments compared to NC as candidate interactors in cases of Level 1, 2, 3, and 4 respectively.

### Bioinformatics analysis

The GO analysis was performed using DAVID Bioinformatics Resources 6.7 (http://david.abcc.ncifcrf.gov) categories Panther_Biological Process (BP)_all^29^. The human genome was used like a background and the level of significance was set to pValue of 10-04 for the Figure 1c. Protein interaction network was generated using the STRING database version 9.0^30^. Visualization in the Figure 1 d,e,f included functional associations and physical interactions which were filtered for medium confidence (>0.400).

### In vitro G3BP1 peptide deacetylation assay

*In vitro* deacetylation reactions were performed with 50 μg of recombinant SIRT6 and 0.5 μg of a peptide corresponding to the G3BP1 sequence acetylated on lysine K376 in the assay buffer (10 mM Tris-HCl pH 8.0, 500 μM NAD^+^, 0.01% BSA, 1 mM DTT, 0.01% DM) at 37°C for 4 h. To detect SIRT6-mediated deacetylation of G3BP1K376ac, mass spectrometry analysis was performed with an Agilent 6130 MSD single quadrupole mass spectrometer, coupled to an Agilent 1200 liquid chromatography system. One hundred microliters of the sample was injected and separated on a reversed-phase chromatography column (ZORBAX Eclipse Plus C18 1.8 μm, 4.6 mm internal diameter 50 mm length, Agilent Technologies) with a linear gradient of acetonitrile in 0.1% trifluoroacetic acid. The flow rate employed was 0.5 mL/min and the column temperature was set at 25°C. Eluted peptides were detected by selected ion monitoring (SIM) equipped with an electrospray ionization source and operated in positive mode (m/z 897.1 (+3) for the acetylated peptide, and m/z 883.1 (+3) for the deacetylated peptide, respectively). Only the m/z value of the acetylated peptide was previously confirmed by using the peptide standard.

### Immunofluorescence and microscopy

U2OS and MEF cells were heat-shocked at 43C for 90 min. Cells were fixed and immunostained as previously described^31^. Confocal images were acquired on Zeiss LSM-510 Meta confocal microscope using a 63 × oil immersion objective and a 512 × 512 pixels resolution. These stacks were then analyzed and stress-granules volume was determined using the 3Dcounter imageJ plugin^32^. Data reported are from 3 independent experiments. For each point in each experiment, at least 100 cells were counted.

### Flow-cytometry

After heat-shock, MEF cells were harvested after 1 h30 or recovered for 24 h at 37C before harvesting. Cells were rinced and resuspended in ice cold Phosphate Buffered Saline (PBS) containing propidium iodide and immediately analyzed by flow cytometry. Flow cytometry was performed on a BD-LSRII cytometer (BD-Bioscience) at the Stanford Shared FACS Facility (SSFF). For detection of propidium iodide staining, sample were excited with a 532 nm Green laser using a 640 a Low-Pass splitter and 670/14 Low-Pass filter. For each sample, 10000 events were collected. Data were analyzed using the flowjo software (Treestar, Inc). Cells stained with propidium iodide were gated (see supplementary Fig. 4) and their percentage compared to the whole population quantified.

## Author Contributions

F.S. and K.F.C. designed the experiments and wrote the manuscript. F.S. performed SIRT6 complexes isolation, bioinformatic analysis, biochemical fractionations, analysis of SIRT6-G3BP1 interaction, heat-shock resistance experiments, microscopy, and flow cytometry. L.T. isolated, characterized, and immortalized WT and SIRT6 KO MEFs, contributed to SIRT6 expression constructs, and together with L.V., contributed to endogenous co-IP studies. S.T., K.K., M.H., E.M.K. and B.A.G. conducted MS analysis. S.T., F.I. and E.M.K. contributed to characterization of G3BP1 complex architecture by siRNA, co-IP experiments, and peptide deacetylation assays.

## Supplementary Material

Supplementary Information Supplementary Information

## References

[b1] Mostoslavsky R. *et al.* Genomic instability and aging-like phenotype in the absence of mammalian SIRT6. Cell 124, 315–329, 10.1016/j.cell.2005.11.044 (2006). 16439206 10.1016/j.cell.2005.11.044

[b2] Michishita E. *et al.* SIRT6 is a histone H3 lysine 9 deacetylase that modulates telomeric chromatin. Nature 452, 492–496, 10.1038/nature06736 (2008). 18337721 10.1038/nature06736 PMC2646112

[b3] Kawahara T. L. *et al.* SIRT6 links histone H3 lysine 9 deacetylation to NF-kappaB-dependent gene expression and organismal life span. Cell 136, 62–74, 10.1016/j.cell.2008.10.052 (2009). 19135889 10.1016/j.cell.2008.10.052 PMC2757125

[b4] Sebastian C. *et al.* The histone deacetylase SIRT6 is a tumor suppressor that controls cancer metabolism. Cell 151, 1185–1199, 10.1016/j.cell.2012.10.047 (2012). 23217706 10.1016/j.cell.2012.10.047 PMC3526953

[b5] Kanfi Y. *et al.* The sirtuin SIRT6 regulates lifespan in male mice. Nature 10.1038/nature10815 (2012). 10.1038/nature10815 22367546

[b6] McCord R. A. *et al.* SIRT6 stabilizes DNA-dependent protein kinase at chromatin for DNA double-strand break repair. Aging 1, 109–121 (2009). 20157594 10.18632/aging.100011 PMC2815768

[b7] Kaidi A., Weinert B. T., Choudhary C. & Jackson S. P. Human SIRT6 promotes DNA end resection through CtIP deacetylation. Science 329, 1348–1353, 10.1126/science.1192049 (2010). 20829486 10.1126/science.1192049 PMC3276839

[b8] Dominy Jr J. E. *et al.* The deacetylase Sirt6 activates the acetyltransferase GCN5 and suppresses hepatic gluconeogenesis. Molecular cell 48, 900–913, 10.1016/j.molcel.2012.09.030 (2012). 23142079 10.1016/j.molcel.2012.09.030 PMC3534905

[b9] Mao Z. *et al.* SIRT6 promotes DNA repair under stress by activating PARP1. Science 332, 1443–1446, 10.1126/science.1202723 (2011). 21680843 10.1126/science.1202723 PMC5472447

[b10] Jiang H. *et al.* SIRT6 regulates TNF-alpha secretion through hydrolysis of long-chain fatty acyl lysine. Nature 496, 110–113, 10.1038/nature12038 (2013). 23552949 10.1038/nature12038 PMC3635073

[b11] Anderson P. & Kedersha N. Stress granules: the Tao of RNA triage. Trends in biochemical sciences 33, 141–150, 10.1016/j.tibs.2007.12.003 (2008). 18291657 10.1016/j.tibs.2007.12.003

[b12] Tourriere H. *et al.* The RasGAP-associated endoribonuclease G3BP assembles stress granules. The Journal of cell biology 160, 823–831, 10.1083/jcb.200212128 (2003). 12642610 10.1083/jcb.200212128 PMC2173781

[b13] Soncini C., Berdo I. & Draetta G. Ras-GAP SH3 domain binding protein (G3BP) is a modulator of USP10, a novel human ubiquitin specific protease. Oncogene 20, 3869–3879, 10.1038/sj.onc.1204553 (2001). 11439350 10.1038/sj.onc.1204553

[b14] Solomon S. *et al.* Distinct structural features of caprin-1 mediate its interaction with G3BP-1 and its induction of phosphorylation of eukaryotic translation initiation factor 2alpha, entry to cytoplasmic stress granules, and selective interaction with a subset of mRNAs. Molecular and cellular biology 27, 2324–2342, 10.1128/MCB.02300-06 (2007). 17210633 10.1128/MCB.02300-06 PMC1820512

[b15] Reineke L. C., Dougherty J. D., Pierre P. & Lloyd R. E. Large G3BP-induced granules trigger eIF2alpha phosphorylation. Molecular biology of the cell 23, 3499–3510, 10.1091/mbc.E12-05-0385 (2012). 22833567 10.1091/mbc.E12-05-0385 PMC3442399

[b16] Tennen R. I., Berber E. & Chua K. F. Functional dissection of SIRT6: identification of domains that regulate histone deacetylase activity and chromatin localization. Mech Ageing Dev 131, 185–192, 10.1016/j.mad.2010.01.006 (2010). 20117128 10.1016/j.mad.2010.01.006 PMC2846990

[b17] Choudhary C. *et al.* Lysine acetylation targets protein complexes and co-regulates major cellular functions. Science 325, 834–840, 10.1126/science.1175371 (2009). 19608861 10.1126/science.1175371

[b18] Wolozin B. Regulated protein aggregation: stress granules and neurodegeneration. Molecular neurodegeneration 7, 56, 10.1186/1750-1326-7-56 (2012). 23164372 10.1186/1750-1326-7-56 PMC3519755

[b19] Gallouzi I. E. Could stress granules be involved in age-related diseases? Aging 1, 753–757 (2009). 20157563 10.18632/aging.100090 PMC2815733

[b20] Aulas A., Stabile S. & Vande Velde C. Endogenous TDP-43, but not FUS, contributes to stress granule assembly via G3BP. Molecular neurodegeneration 7, 54, 10.1186/1750-1326-7-54 (2012). 23092511 10.1186/1750-1326-7-54 PMC3502460

[b21] Chiang W. C. *et al.* C. elegans SIRT6/7 homolog SIR-2.4 promotes DAF-16 relocalization and function during stress. PLoS Genet 8, e1002948, 10.1371/journal.pgen.1002948 (2012). 23028355 10.1371/journal.pgen.1002948 PMC3441721

[b22] Michishita E., Park J. Y., Burneskis J. M., Barrett J. C. & Horikawa I. Evolutionarily conserved and nonconserved cellular localizations and functions of human SIRT proteins. Molecular biology of the cell 16, 4623–4635 (2005). 16079181 10.1091/mbc.E05-01-0033 PMC1237069

[b23] Dignam J. D., Lebovitz R. M. & Roeder R. G. Accurate transcription initiation by RNA polymerase II in a soluble extract from isolated mammalian nuclei. Nucleic acids research 11, 1475–1489 (1983). 6828386 10.1093/nar/11.5.1475 PMC325809

[b24] Mendez J. & Stillman B. Chromatin association of human origin recognition complex, cdc6, and minichromosome maintenance proteins during the cell cycle: assembly of prereplication complexes in late mitosis. Molecular and cellular biology 20, 8602–8612 (2000). 11046155 10.1128/mcb.20.22.8602-8612.2000 PMC102165

[b25] Wessel D. & Flugge U. I. A method for the quantitative recovery of protein in dilute solution in the presence of detergents and lipids. Analytical biochemistry 138, 141–143 (1984). 6731838 10.1016/0003-2697(84)90782-6

[b26] Rappsilber J., Mann M. & Ishihama Y. Protocol for micro-purification, enrichment, pre-fractionation and storage of peptides for proteomics using StageTips. Nature Protocols 2, 1896–1906 (2007). 10.1038/nprot.2007.261 17703201

[b27] Huttlin E. L. *et al.* A tissue-specific atlas of mouse protein phosphorylation and expression. Cell 143, 1174–1189, 10.1016/j.cell.2010.12.001 (2010). 21183079 10.1016/j.cell.2010.12.001 PMC3035969

[b28] Elias J. E. & Gygi S. P. Target-decoy search strategy for increased confidence in large-scale protein identifications by mass spectrometry. Nature methods 4, 207–214, 10.1038/nmeth1019 (2007). 17327847 10.1038/nmeth1019

[b29] Huang da W., Sherman B. T. & Lempicki R. A. Systematic and integrative analysis of large gene lists using DAVID bioinformatics resources. Nature protocols 4, 44–57, 10.1038/nprot.2008.211 (2009). 10.1038/nprot.2008.211 19131956

[b30] Szklarczyk D. *et al.* The STRING database in 2011: functional interaction networks of proteins, globally integrated and scored. Nucleic acids research 39, D561–568, 10.1093/nar/gkq973 (2011). 21045058 10.1093/nar/gkq973 PMC3013807

[b31] Kedersha N., Tisdale S., Hickman T. & Anderson P. Real-time and quantitative imaging of mammalian stress granules and processing bodies. Methods in enzymology 448, 521–552, 10.1016/S0076-6879(08)02626-8 (2008). 19111193 10.1016/S0076-6879(08)02626-8

[b32] Bolte S. & Cordelieres F. P. A guided tour into subcellular colocalization analysis in light microscopy. Journal of microscopy 224, 213–232, 10.1111/j.1365-2818.2006.01706.x (2006). 17210054 10.1111/j.1365-2818.2006.01706.x

