# Supplementary material for: Proteomic analysis of the SIRT6 interactome: novel links to genome maintenance and cellular stress signaling
Source: Sci Rep. 2013 Oct 30;3:3085. doi: 10.1038/srep03085 (PMC3812651; doi:10.1038/srep03085)
Supplement: Supplementary Information [file srep03085-s1.pdf]

**Supplementary Information**

# Proteomic analysis of the SIRT6 interactome: novel links to genome maintenance and cellular stress signaling

Federica Simeoni [1,2], Luisa Tasselli [1,2], Shinji Tanaka [3,7], Lidia Villanova [1,2,4], Mayumi Hayashi [5], Kazuishi Kubota [5], Fujio Isono [3], Benjamin A. Garcia [6], Eriko Michishita-Kioi [3,7] *, Katrin F. Chua[1,2,*]

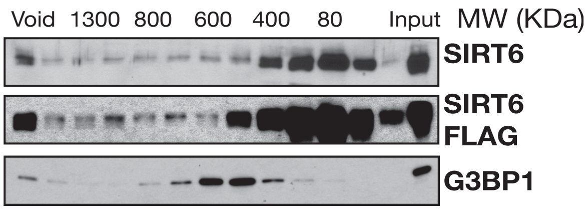


**Supplementary figure 1.** Elution profiles of endogenous SIRT6, SIRT6-FLAG, and endogenous G3BP1 in gel filtration fractions (Superose 6) of nuclear extracts. Endogenous SIRT6 and SIRT6-FLAG from HeLa S3 nuclear extract show the same elution profile. A subset of SIRT6 co-fractionates with the G3BP1 peak at 600kDa. Alternate (0.5 ml) fractions between void volume and 40 KDa were collected and analyzed. Nuclear extract from HeLa S3 cells were used for detection of endogenous SIRT6, while nuclear extract from HeLa S3 cells stably expressing SIRT6-FLAG were used for detection of SIRT6-FLAG and G3BP1 by western blot.

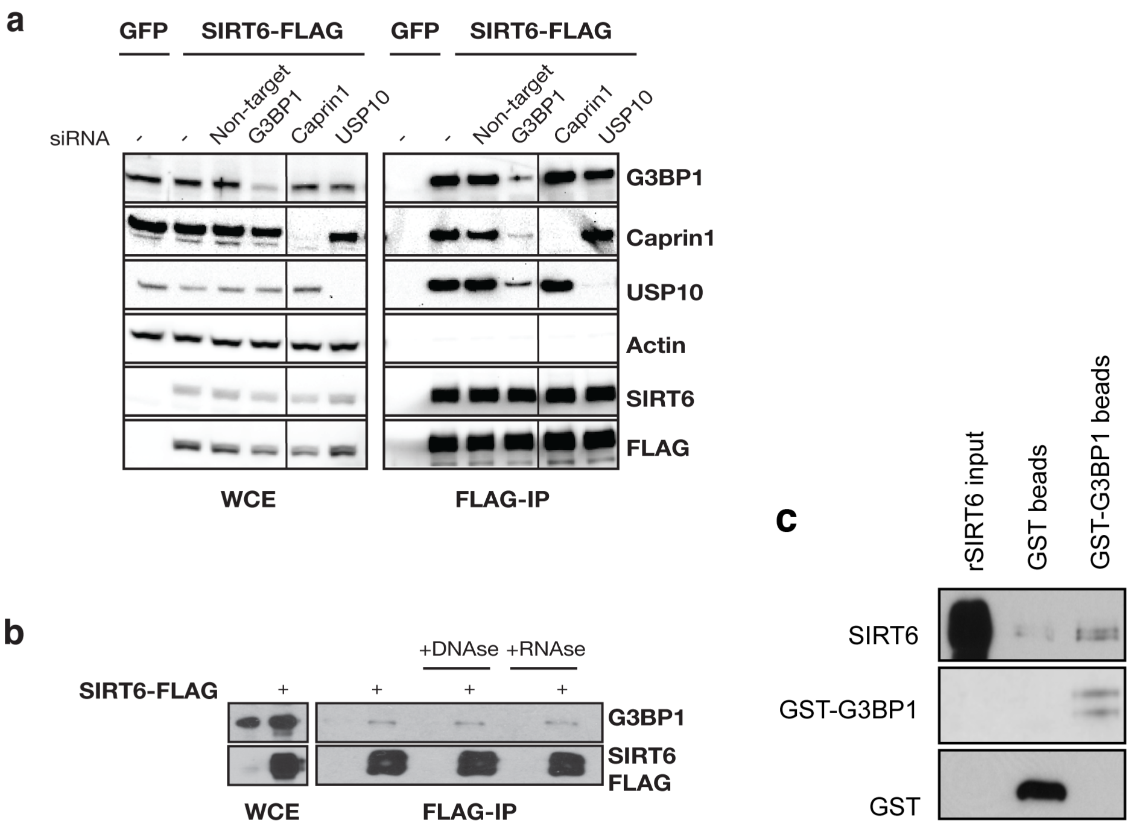


**Supplementary Figure 2.** Biochemical characterization of SIRT6-G3BP1 interaction. **(a)** 293T cells were transfected with siRNAs targeting G3BP1, Caprin1, USP10, or control sequence, and after 24 hrs, with FLAG-tagged human SIRT6 or GFP control. 48 hrs after plasmid transfection, FLAG-tagged proteins were immunoprecipitated and analyzed by western blot for G3BP1, Caprin1, USP10, SIRT6 and actin. **(b)** Nuclear extract lysates from HeLa S3 cells stably expressing SIRT6-FLAG or empty vector control were incubated with DNase and RNase, immunoprecipitated and analyzed for the presence of G3BP1 and FLAG by western blot. **(c)** In vitro pull-down assays of recombinant purified SIRT6 by purified GST-SIRT6 or GST control proteins. Lines indicate cropped parts of the same gel, where irrelevant lanes were eliminated.

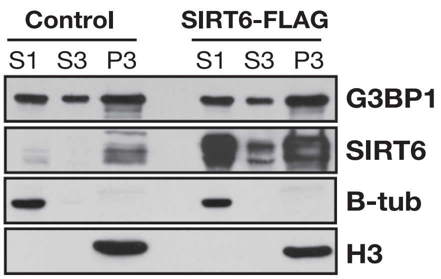


**Supplementary Figure 3.** G3BP1 is a chromatin bound protein. HeLa S3 cells stably expressing SIRT6-FLAG or empty vector control were biochemically fractionated as previously described (7)**.** Fractions: S1, cytoplasm; S3, soluble nuclear; P3, chromatin enriched proteins.

‘

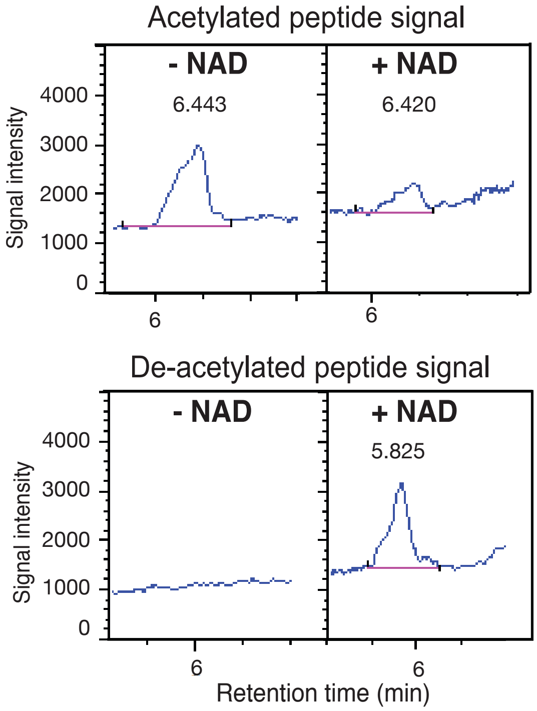


**Supplementary Figure 4.** SIRT6 deacetylates G3BP1(K376Ac) peptide *in vitro*. (**a)** Recombinant SIRT6 was incubated in presence or absence of NAD+ with the G3BP1-(K376Ac) peptide. Retention time of the acetylated and deacetylated G3BP1 peptide are respectively 6.4 and 5.8 min.

**Supplementary Table 1.** Protein enrichment by Gel Filtration Chromatography (GFC) followed by SIRT6-FLAG pulldown. Comparison of PSMs (peptide-spectrum matches) of proteins identified in SIRT6-FLAG versus Negative Control (NC) pulldown. From 2 independent experiments (1st and 2nd MS analysis), in total, 85 proteins were reproducibly enriched in SIRT6-Flag IPs from the same fractions of Gel Filtration Chromatography (size range: 1300-800 KDa, 800-600 KDa, 600-400 KDa).

# Reproducibility of Gel Filtration Chromatography (GFC) samples (1st and 2nd MS analyses)

1st MS analysis

Peptide–spectrum match (PSM)

| | | Experiment A GFC | | | | | |
|---|---|---|---|---|---|---|---|
| | | NC | | | S6 | | |
| | | 1300_800kD | 800_600kDa | 600_400kDa | 1300_800kD | 800_600kDa | 600_400kDa |
| reference | Gene symbol | | | | | | |
| Q8N6T7 | SIRT6 | 7 | | 3 | 397 | 452 | 713 |
| P07355 | ANXA2 | 10 | 2 | 52 | 25 | 40 | 96 |
| P61978 | HNRNPK | | 3 | 38 | 26 | 36 | 75 |
| P13639 | EEF2 | | | 2 | | 5 | 22 |
| Q86UV5 | USP48 | | | 2 | | | 14 |
| Q96HC4 | PDLIM5 | | | | | | 13 |
| P68104 | EEF1A1 | 13 | 13 | 57 | 70 | 91 | 135 |
| P11142 | HSPA8 | 2 | 3 | 11 | 61 | 60 | 50 |
| P49005 | POLD2 | | | | | 18 | 34 |
| P04406 | GAPDH | 5 | 2 | 3 | 4 | 18 | 6 |
| P54652 | HSPA2 | | | 3 | 13 | 15 | 14 |
| P34931 | HSPA1L | 2 | 2 | 8 | 12 | 15 | 17 |
| Q9BR76 | CORO1B | | | 3 | | | 14 |
| P06748 | NPM1 | | | 3 | 12 | 23 | 29 |
| Q71U36 | TUBA1A | 3 | 1 | 3 | 74 | 63 | 57 |
| Q13885 | TUBB2A | | | 3 | 56 | 55 | 64 |
| P43246 | MSH2 | | | 10 | 2 | 23 | 53 |
| P06702 | S100A9 | 2 | 2 | | 4 | 33 | |
| Q16531 | DDB1 | | 1 | 3 | 10 | 45 | 33 |
| Q15054 | POLD3 | | | | | 5 | 12 |
| Q13283 | G3BP1 | | | | 12 | 23 | 5 |
| P43243 | MATR3 | | 3 | 3 | 33 | 63 | 37 |
| P26641 | EEF1G | 21 | 15 | 7 | 75 | 29 | 20 |
| P52701 | MSH6 | | | 2 | 8 | 33 | 44 |
| Q14444 | CAPRIN1 | | | | 16 | 11 | |
| O14744 | PRMT5 | 13 | 68 | 27 | 97 | 221 | 67 |
| P02545 | LMNA | 20 | 2 | 1 | 122 | 41 | 5 |
| P12814 | ACTN1 | | 8 | | 26 | 122 | 6 |
| P15924 | DSP | 53 | 4 | 9 | 12 | 21 | 1 |
| P53396 | ACLY | | | 16 | 2 | 82 | 96 |

| | | | | | | | |
|---|---|---|---|---|---|---|---|
| P78347 | GTF2I | 6 | 10 | | 77 | 40 | 5 |
| Q93009 | USP7 | | 4 | 27 | 9 | 49 | 41 |
| O00267 | SUPT5H | | | | | 17 | 40 |
| Q5VTR2 | RNF20 | | | | 34 | 8 | |
| P12268 | IMPDH2 | | | 2 | | 7 | 34 |
| Q14839 | CHD4 | 3 | | | 33 | 2 | |
| Q8N163 | KIAA1967 | | | | 30 | | |
| Q13263 | TRIM28 | | | | 29 | 7 | |
| O75150 | RNF40 | | | | 28 | 9 | |
| P05109 | S100A8 | 3 | 4 | 2 | 5 | 20 | 4 |
| P50990 | CCT8 | | | | 7 | 12 | |
| Q14694 | USP10 | | | | 1 | 10 | |
| O95239 | KIF4A | | | | 12 | 22 | |
| O75717 | WDHD1 | | | | 21 | 3 | |
| Q8WX93 | PALLD | | | 3 | | 3 | 18 |
| Q15021 | NCAPD2 | | | | 17 | 3 | |
| P47756 | CAPZB | | | | 17 | 10 | |
| Q7Z5K2 | WAPAL | | | | 16 | 14 | |
| Q15029 | EFTUD2 | | | | 16 | 3 | |
| Q14258 | TRIM25 | | | | | 15 | 11 |
| P62333 | PSMC6 | | | | 14 | | |
| P17987 | TCP1 | | | | 4 | 13 | |
| P52907 | CAPZA1 | | | | 12 | 6 | |
| Q15020 | SART3 | | | | 12 | | |
| P12035 | KRT3 | 5 | 1 | | | 7 | |
| Q4G0J3 | LARP7 | | | | 10 | | |
| P40222 | TXLNA | | | | 9 | 1 | |
| P48643 | CCT5 | | | | | 7 | |
| Q9BTA9 | WAC | | | | 6 | | |
| O60506 | SYNCRIP | | | | 6 | 10 | 5 |
| P31943 | HNRNPH1 | 3 | 10 | 13 | 18 | 28 | 22 |
| P78527 | PRKDC | 10 | | | 456 | 140 | 14 |
| P21333 | FLNA | 33 | 3 | | 258 | 39 | 5 |
| O75369 | FLNB | 22 | | | 244 | 9 | |
| Q09028 | RBBP4 | 5 | 2 | 3 | 22 | 4 | |
| Q92769 | HDAC2 | 4 | | | 20 | 5 | 2 |
| O94776 | MTA2 | 2 | | | 30 | 5 | 1 |
| Q14683 | SMC1A | | | | 56 | 4 | |
| O95347 | SMC2 | | | | 41 | 3 | |
| Q9NTJ3 | SMC4 | | | | 48 | 7 | |

| | | | | | | | |
|---|---|---|---|---|---|---|---|
| Q9UQE7 | SMC3 | | | | 46 | 2 | |
| Q29RF7 | PDS5A | | | | 9 | 9 | 2 |
| Q9Y265 | RUVBL1 | | | | 43 | 34 | 7 |
| Q9Y230 | RUVBL2 | | | | 64 | 38 | 16 |
| O75643 | SNRNP200 | | | | 31 | 8 | 2 |
| Q13428 | TCOF1 | | | | 6 | 5 | |
| Q16576 | RBBP7 | | | | 31 | 8 | 2 |
| O95071 | UBR5 | | | | 10 | | |
| O60216 | RAD21 | | | | 11 | 1 | |
| P30260 | CDC27 | | | | 17 | | |
| Q8WXI9 | GATAD2B | | | | 16 | | |
| Q9UJX3 | ANAPC7 | | | | 29 | 7 | 1 |
| Q13547 | HDAC1 | | | | 25 | 8 | |
| Q6P2Q9 | PRPF8 | | | | 26 | 2 | |
| Q92878 | RAD50 | | | | 14 | | |
| Q9BZK7 | TBL1XR1 | | | | 13 | | |
| Q9H1A4 | ANAPC1 | | | | 20 | | |

**2nd MS analysis**

Peptide-spectrum match (PSM)

| | | Experiment B GFC | | | | | |
|---|---|---|---|---|---|---|---|
| | | NC | | | S6 | | |
| | | 1300_800kD | 800_600kDa | 600_400kDa | 1300_800kD | 800_600kDa | 600_400kDa |
| reference | Gene symbol | | | | | | |
| Q8N6T7 | SIRT6 | 6 | | | 324 | 259 | 363 |
| P07355 | ANXA2 | 10 | 3 | 6 | 21 | 27 | 26 |
| P61978 | HNRNPK | | | | 18 | 11 | 30 |
| P13639 | EEF2 | | | | | 2 | 23 |
| Q86UV5 | USP48 | | | | | | 10 |
| Q96HC4 | PDLIM5 | | | | | | 6 |
| P68104 | EEF1A1 | 7 | 6 | | 40 | 37 | 36 |
| P11142 | HSPA8 | | | | 15 | 12 | 22 |
| P49005 | POLD2 | | | | | 2 | 37 |
| P04406 | GAPDH | 6 | | 3 | 1 | 15 | 12 |
| P54652 | HSPA2 | | | | 5 | 8 | 8 |
| P34931 | HSPA1L | | | | 8 | 3 | 3 |
| Q9BR76 | CORO1B | | | | | | 10 |
| P06748 | NPM1 | | | | 8 | 18 | 20 |
| Q71U36 | TUBA1A | | | | 3 | 10 | 4 |
| Q13885 | TUBB2A | | | | 5 | 4 | 6 |

| | | | | | | | |
|---|---|---|---|---|---|---|---|
| P43246 | MSH2 | | | | 4 | 10 | 19 |
| P06702 | S100A9 | 17 | 3 | 5 | 9 | 49 | 13 |
| Q16531 | DDB1 | | | | 8 | 10 | 7 |
| Q15054 | POLD3 | | | | | | 22 |
| Q13283 | G3BP1 | | | | 16 | 40 | 30 |
| P43243 | MATR3 | | | | 16 | 54 | 94 |
| P26641 | EEF1G | 2 | 1 | | 45 | 13 | 7 |
| P52701 | MSH6 | | | | 4 | 5 | 22 |
| Q14444 | CAPRIN1 | | | | 15 | 28 | 5 |
| O14744 | PRMT5 | 4 | 9 | | 32 | 46 | 17 |
| P02545 | LMNA | 4 | | | 108 | 18 | 1 |
| P12814 | ACTN1 | | | | 3 | 17 | |
| P15924 | DSP | 109 | 44 | 39 | 51 | 112 | 63 |
| P53396 | ACLY | | | | | 18 | 26 |
| P78347 | GTF2I | | | | 28 | 17 | |
| Q93009 | USP7 | | | | 6 | 11 | 23 |
| O00267 | SUPT5H | | | | | | 12 |
| Q5VTR2 | RNF20 | | | | 38 | 6 | |
| P12268 | IMPDH2 | | | | | 2 | 7 |
| Q14839 | CHD4 | | | | 9 | | |
| Q8N163 | KIAA1967 | | | | 30 | | |
| Q13263 | TRIM28 | | | | 16 | 7 | |
| O75150 | RNF40 | | | | 20 | | |
| P05109 | S100A8 | 17 | 3 | 4 | 5 | 25 | 13 |
| P50990 | CCT8 | | | | 6 | 25 | 2 |
| Q14694 | USP10 | | | | 3 | 24 | 7 |
| O95239 | KIF4A | | | | 9 | 10 | |
| O75717 | WDHD1 | | | | 9 | 2 | |
| Q8WX93 | PALLD | | | | | | 9 |
| Q15021 | NCAPD2 | | | | 17 | | |
| P47756 | CAPZB | | | | 6 | | |
| Q7Z5K2 | WAPAL | | | | 12 | | |
| Q15029 | EFTUD2 | | | | 11 | | |
| Q14258 | TRIM25 | | | | 4 | 15 | 5 |
| P62333 | PSMC6 | | | | 7 | | |
| P17987 | TCP1 | | | | 1 | 12 | |
| P52907 | CAPZA1 | | | | 9 | 2 | |
| Q15020 | SART3 | | | | 10 | | |
| P12035 | KRT3 | 7 | | | 5 | 10 | 3 |
| Q4G0J3 | LARP7 | | | | 9 | | |

| | | | | | |
|---|---|---|---|---|---|
| P40222 | TXLNA | | 7 | | |
| P48643 | CCT5 | | 1 | 7 | 1 |
| Q9BTA9 | WAC | | 7 | | |
| O60506 | SYNCRIP | | 6 | 4 | 1 |
| P31943 | HNRNPH1 | | 12 | 5 | 15 |
| P78527 | PRKDC | | 81 | 70 | 80 |
| P21333 | FLNA | 48 | 161 | 8 | |
| O75369 | FLNB | | 125 | | |
| Q09028 | RBBP4 | | 8 | 2 | |
| Q92769 | HDAC2 | | 7 | | |
| O94776 | MTA2 | | 26 | | |
| Q14683 | SMC1A | | 57 | 9 | |
| O95347 | SMC2 | | 60 | 5 | |
| Q9NTJ3 | SMC4 | | 58 | 6 | |
| Q9UQE7 | SMC3 | | 53 | 3 | |
| Q29RF7 | PDS5A | | 37 | 2 | |
| Q9Y265 | RUVBL1 | | 21 | 7 | 4 |
| Q9Y230 | RUVBL2 | | 13 | 5 | 2 |
| O75643 | SNRNP200 | | 17 | | |
| Q13428 | TCOF1 | | 13 | | |
| Q16576 | RBBP7 | | 13 | | |
| O95071 | UBR5 | | 12 | | |
| O60216 | RAD21 | | 11 | | |
| P30260 | CDC27 | | 11 | | |
| Q8WXI9 | GATAD2B | | 11 | | |
| Q9UJX3 | ANAPC7 | | 11 | | |
| Q13547 | HDAC1 | | 10 | | |
| Q6P2Q9 | PRPF8 | | 8 | | |
| Q92878 | RAD50 | | 7 | | |
| Q9BZK7 | TBL1XR1 | | 6 | | |
| Q9H1A4 | ANAPC1 | | 6 | | |

**Supplementary Table 2.** SIRT6-FLAG interacting proteins in the three size ranges (1300-800 KDa, 800-600 KDa, 600-400 KDa) of Gel Filtration Chromatography.
Gene Ontology (GO) was performed using DAVID, categories Panther_BP_all. The human genome was used as background.

List of SIRT6 Interactors enriched in specific fractions after Gel Filtration Chromatography.

| 1300-800KDa | 800-600KDa | 600-400KDa |
|---|---|---|
| HNRNPK | HNRNPK | EEF1A1 |
| EEF1A1 | EEF1A1 | HSPA8 |
| HSPA8 | HSPA8 | NPM1 |
| HSPA1L | NPM1 | DDB1 |
| NPM1 | DDB1 | MATR3 |
| DDB1 | G3BP1 | PRMT5 |
| G3BP1 | MATR3 | PRKDC |
| MATR3 | CAPRIN1 | HSPA2 |
| EEF1G | PRMT5 | MSH2 |
| CAPRIN1 | LMNA | ACLY |
| PRMT5 | GTF2I | TUBB2A |
| LMNA | USP7 | MSH6 |
| GTF2I | RNF20 | POLD2 |
| USP7 | TRIM28 | SUPT5H |
| RNF20 | CCT8 | IMPDH2 |
| CHD4 | KIF4A | EEF2 |
| KIAA1967 | PRKDC | USP48 |
| TRIM28 | FLNA | PDLIM5 |
| RNF40 | SMC4 | CORO1B |
| CCT8 | RUVBL1 | POLD3 |
| KIF4A | HSPA2 | PALLD |
| WDHD1 | TUBA1A | |
| NCAPD2 | ACTN1 | |
| CAPZB | ANXA2 | |
| WAPAL | GAPDH | |
| EFTUD2 | MSH2 | |
| PSMC6 | S100A9 | |
| CAPZA1 | DSP | |
| SART3 | ACLY | |
| LARP7 | S100A8 | |
| TXLNA | USP10 | |
| WAC | TRIM25 | |
| SYNCRIP | TCP1 | |
| HNRNPH1 | KRT3 | |
| PRKDC | CCT5 | |
| FLNA | | |
| FLNB | | |
| RBBP4 | | |
| HDAC2 | | |
| MTA2 | | |
| SMC1A | | |
| SMC2 | | |
| SMC4 | | |
| SMC3 | | |
| PDS5A | | |
| RUVBL1 | | |
| RUVBL2 | | |

List of SIRT6 Interactors enriched in specific fractions after Gel Filtration Chromatography.

SNRNP200
TCOF1
RBBP7
UBR5
RAD21
CDC27
GATAD2B
ANAPC7
HDAC1
PRPF8
RAD50
TBL1XR1
ANAPC1
